# Relationship between seedling and mature vegetation on the hilly-gullied Loess Plateau

**DOI:** 10.1186/2193-1801-2-S1-S17

**Published:** 2013-12-11

**Authors:** Yan-feng Jia, Ju-ying Jiao, Ning Wang

**Affiliations:** Institute of Soil and Water Conservation, Chinese Academy of Sciences and Ministry of Water Resources, Yangling, Shaanxi 712100 China; Institute of Soil and Water Conservation, Northwest A&F University, Yangling, Shaanxi 712100 China; Graduate School of Chinese Academy of Sciences, Beijing, China

**Keywords:** Seedling density, Seedling species, Diversity, Similarity

## Abstract

Seedling is an indispensable stage in plant cycle life, and seedling survival is important during natural vegetation restoration, especially on the Loess Plateau. In 2007, we selected 4 plots of *Artemisia scoparia* communities (ASC) and 4 plots of *Artemisia gmelinii* + *Artemisia giraldii* communities (AGC), examined seedling richness, diversity during the rainy season, and examined mature vegetation richness, coverage, and frequency in August. The results showed that seedlings density of ASC were 29 n m^-2^, 33 n m^-2^, 20 n m^-2^ and 31 n m^-2^ in July to October respectively, and that of AGC were 14 n m^-2^, 12 n m^-2^, 6 n m^-2^ and 9 n m^-2^ respectively; *A. scoparia* seedlings represented 53.2% of the total seedlings in ASC, the dominant species in AGC only account for less than 5% of the total seedlings. Most of the seedlings found were belonged to *Compositae, Leguminoseae* and *Gramineae*; 80% of seedlings in ASC were mainly comprised of *A. scoparia* and *Lespedeza davurica*, while in AGC that consisted of more than 6 species, such as *L. davurica, Sophora viciifolia, Dracocephalum moldavicaand, A. gmelinii, Patrinia heterophylla, Heteropappus altaicus* so on. Sørensen similarity index between monthly seedlings was approximately 0.47 in ASC and 0.35 in AGC; Sørensen similarity index between seedlings and mature vegetation ranged from 0.18 to 0.34 in ASC, and varied from 0.26 to 0.39 in AGC. These results suggested that seedling establishment would be a bottleneck for natural vegetation restoration when seed supply and seedling emergence were possible.

## Introduction

The Loess Plateau is well known as one of the most rapidly eroding areas in the world, with average soil losses of 3,720 t km^-2^ a^-1^ [1]. For such a serious soil erosion area, reducing soil erosion is an important and urgent issue. Vegetation is one of the most efficient methods to control soil loss and ameliorate ecological environment [2, 3]. However, widespread tree planting just retained significant tree cover in less than 10% of the total area [4]. Furthermore, planted trees and grasses take up soil water from deep soil layers and cause soil desiccation in the longer-term [5, 6]. The restoration of nature vegetation is the most important measure to improve the ecological conditions on the Loess Plateau [7].

Sufficient seed in the soil seed back is important for recovery, but the germination of seeds and survival of seedlings are constrained by several environment factors [8]. In the hilly-gullied Loess Plateau, the seed density of soil seed bank ranged from 1,067 seeds m^-2^ to 14,967 seeds m^-2^ in 0-10 cm layer [9, 10], it is within the range reported by other studies on the grassland in the United States, Canada, Europe, and Japan with seed bank density varied from 287 seeds m^-2^ to 31 344 seeds m^-2^ [11, 12]. Seed density of the soil seed bank in the hilly-gullied Loess Plateau represented a medium level of soil seed bank, and concluded that the seed bank is large enough to allow natural restoration [13].

However, in the life history of plants, seed and seedling stages are particularly vulnerable to environmental conditions [14, 15]. The dynamic of these stages influence the structure of both adult populations and communities [16]. And seedling recruitment represents a bottleneck for most populations [17]. Recruitment dynamics can be a key determinant of plant population growth and persistence [18], the success of regeneration of natural vegetation largely depends on the success of seedling survival and establishment, especially in harsh environment. And the aim of this study was to check: (1) differences in seedling density and species of different communities, (2) the similarity between seedlings in the continuous months to evaluate the seedling persistence, (3) the similarity between seedlings and the standing vegetation to evaluate the contribution of seedlings made to the mature vegetation.

## Method

### Study site

Zhifanggou watershed, located in the northern Shaanxi province, was selected as study site (E109°13ʹ46ʺ-109°16ʹ03ʺ, N36°42ʹ42ʺ-36°46ʹ28ʺ). The elevation ranges from 1,041.5 m to 1,425.71 m and 80.29% of the slopes have a gradient greater than 15° [19]. The climate is characterized by cold, dry winters and warm, moist summers, and the mean temperature is approximately 8.8 °C. The average annual precipitation is approximately 500 mm, and 74% of rainfall occurs between June and September. The study area is in the temperate forest-steppe zone [20], but due to long-term human activity, most of the natural vegetation has been replaced by cultivated land for maize (*Zea mays*), millet (*Panicum miliaceum*) and potatoes (*Solanum tuberosum*) since the 1950s [21, 22]. The forests are dominated by artificial *Robinia pseudoacacia*, and the shrubland is dominated by artificial *Caragana korshinskii*, artificial *Hippophae rhamnoides* and natural *Sophora viciifolia*. The steppe vegetation is mainly comprised of *Artemisia gmelinii, Artemisia giraldii, Lespedeza davurica* and *Stipa bungeana* [23].

### Vegetation analysis

Field investigations were conducted in 2007. We selected two typical communities during natural succession process for investigation; one was *Artemisia scoparia* community (ASC) in early stage and another was *A. gmelinii* + *A. giraldii* community (AGC) in later stage, and 4 plots in each type were used. The quadrates size was 50 cm × 50 cm for seedlings and 1 m × 1 m for mature vegetation. During the rainy season (from July to October), seedlings richness and diversity were examined each month, and mature vegetation richness, coverage, and frequency were examined in August. Then we compared the seedlings density and seedling composition of each month with that of next month, and compared the seedling composition with that of mature vegetation.

The diversity indexes were calculated using the following formulas [24]:

Where *d*_*Ma*_ is Margalef index, *H'* is Shannon-Wiener index, *J*_*sw*_ is Pielou index, *N* is the total number of individuals, *P*_*i*_ is the number of individuals of one species in relation to the number of individuals in the population, *S* is the total number of species in the sample.

Sørenson's index of similarity [25] was used to calculate the similarity between monthly seedlings, and the similarity between seedlings and the standing vegetation:

Where *CC* is Sørenson similarity, *A* and *B* are the number of all the species found in the two samples, and *W* is the number of species found in both samples.

## Results

### Seedlings density

**Figure 1:**
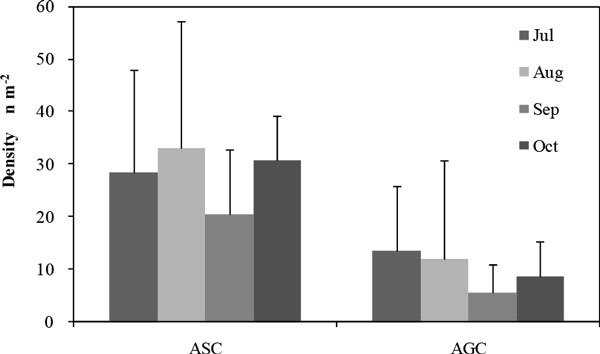
**Seedlings density of ASC and AGC**.

Seedlings in ASC were more than that in AGC in each month, while significant different only arisen in August and September (Figure 1). From July to October, seedlings of ASC were 29 n m^-2^, 33 n m^-2^, 20 n m^-2^ and 31 n m^-2^, respectively, which were most in August and least in September; while seedlings of AGC were most in July and least in September, and seedlings densities were 14 n m^-2^, 12 n m^-2^, 6 n m^-2^ and 9 n m^-2^, respectively. But there was no significant difference between months in each community.

**Figure 2:**
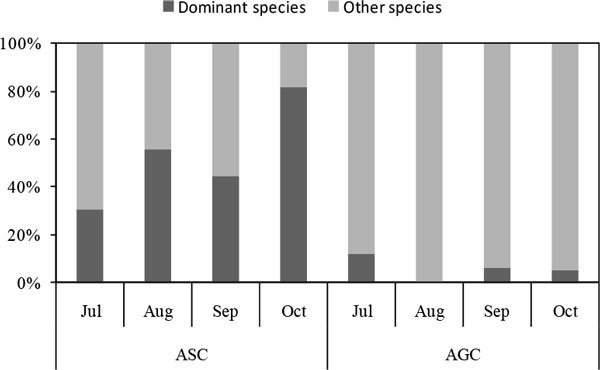
**Composition of seedlings in ASC and AGC**. Note: In ASC, dominant species refers to *Artemisia scoparia*, and other species refer to *Lespedeza davurica, Heteropappus altaicus, Ixeris Chinensis*, and so on. In AGC, dominant species refers to *Artemisia gmelinii* and *Artemisia giraldii*, and other species refer to *Lespedeza davurica, Sophora viciifolia, Dracocephalum moldavicaand, Heteropappus altaicus, Patrinia heterophylla, Vicia amoena* and so on.

From July to October, the dominant species *A. scoparia* with seedling density of 9 n m^-2^, 19 n m^-2^, 9 n m^-2^ and 25 n m^-2^ , respectively, which represented 30.4%, 55.8%, 44.9% and 81.6% of the total seedlings in ASC. In AGC, the dominant species *A. gmelinii* appeared in July, September and October with density of 1.5 n m^-2^, 0.3 n m^-2^ and 0.3 n m^-2^, respectively; seedlings of the other dominant species *A. giraldii* just were found in July and October with density of 0.2 n m^-2^ and 0.2 n m^-2^, respectively. Both of the dominant species seedlings represented less than 10% of the total seedlings in each month (Figure 2).

These were consistent with the composition of the soil seed bank. In soil seed bank, *A. scoparia* had the biggest density of 6 888.7 seed m^-2^, which could take approximately 84.8% of the total seed in the soil seed bank. *A. gmelinii* with lower density of approximately 297.5 seed m^-2^ accounted for 2% of the total seed in soil seed bank, and *A. giraldii* with density of approximately 103.3 seed m^-2^ only accounted for 0.7% of the total seed in soil seed bank [26].

### Seedlings species and seedlings diversity

From July to October, 22 species seedlings in 9 families were found in ASC, most of them were belonged to *Compositae* (6 species), *Gramineae* (5 species) and *Leguminoseae* (3 species); and 32 species in 13 families were found in AGC, 50% of them were contained in *Compositae* (9 species) and *Leguminoseae* (7 species).

Seedlings of ASC were mainly composited by *A. scoparia* and *L. davurica*, and the first two main species constituted 64.6% to 89.7% of the total seedlings; while 80% of seedlings in AGC would consist of more than 6 species, such as *L. davurica, S. viciifolia, Dracocephalum moldavicaand, A. gmelinii, Patrinia heterophylla, Heteropappus altaicus* and so on (Table 1).

**Table 1:** Seedlings species of ASC and AGC

Types	Mouth	Three main species (%)
	Jul	*Lespedeza davurica* (34.2%) *Artemisia scoparia* (30.4%) *Potentilla tanacetifolia* (7.9%)
ASC	Aug	*Artemisia scoparia* (55.8%) *Lespedeza davurica* (30.4%) *Ixeris Chinensis* (3.8%)
	Sep	*Lespedeza davurica* (40.8%) *Artemisia scoparia* (44.9%) *Lappula myosotis* (6.9%)
	Oct	*Artemisia scoparia* (81.6%) *Lespedeza davurica* (8.1%) *Heteropappus altaicus* (3.0%)
	Jul	*Cleistogenes squarrosa* (21.3%) *Lespedeza davurica* (18.1%) *Artemisia gmelinii* (11.3%)
AGC	Aug	*Sophora viciifolia* (71.9%) *Lespedeza davurica* (18.3%) *Dracocephalum moldavicaand* (2.8%)
	Sep	*Lespedeza davurica* (29.9%) *Dracocephalum moldavicaand* (11.9%) *Astragalus scaberrimus* (11.9%)
	Oct	*Lespedeza davurica* (10.8%) *Ixeris Chinensis* (18.6%) *Artemisia scoparia* (5.9%)

**Figure 3:**
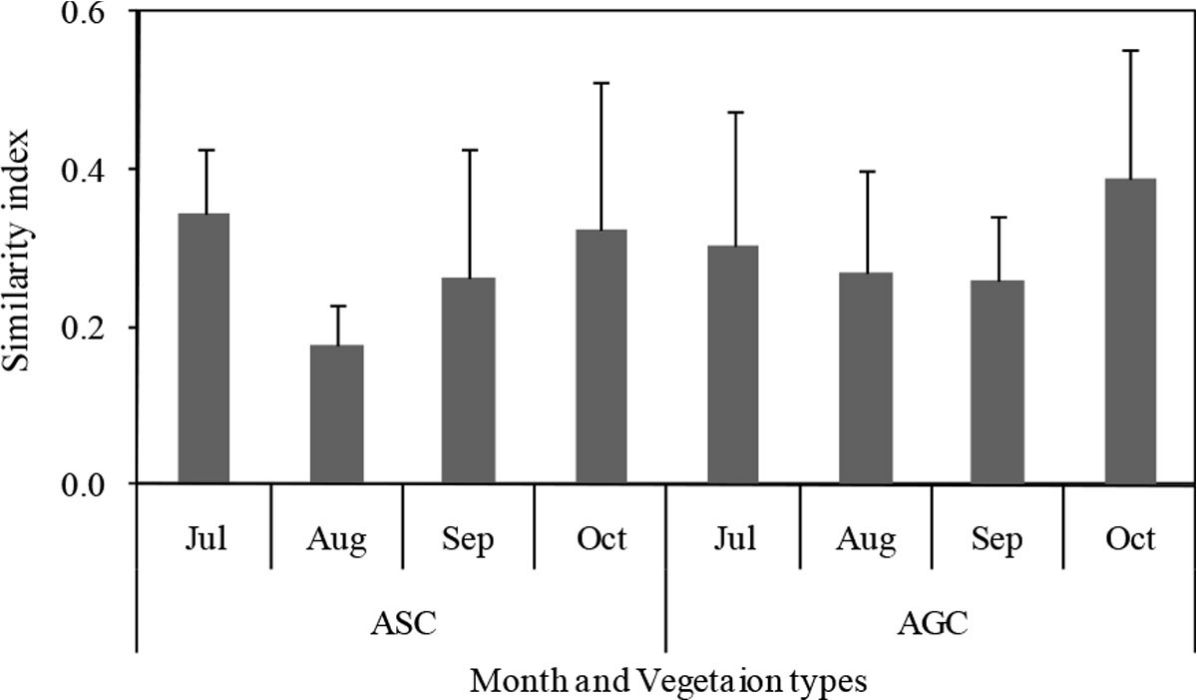
**Seedlings diversity of ASC and AGC**.

Seedlings diversity also suggested that AGC exhibited higher species richness. Diversity indexes of AGC were always higher than that of ASC (Figure 3). For example, in July, Margalef index, Pielou index, and Shannon-wiener index of ASC were 2.69, 0.75 and 1.72, of AGC was 6.56, 0.81 and 2.33, and in October, the three indexes of ASC were 2.62, 0.34 and 0.78, and in AGC were 8.88, 0.90 and 2.70.

### Similarity between monthly seedlings

**Figure 4:**
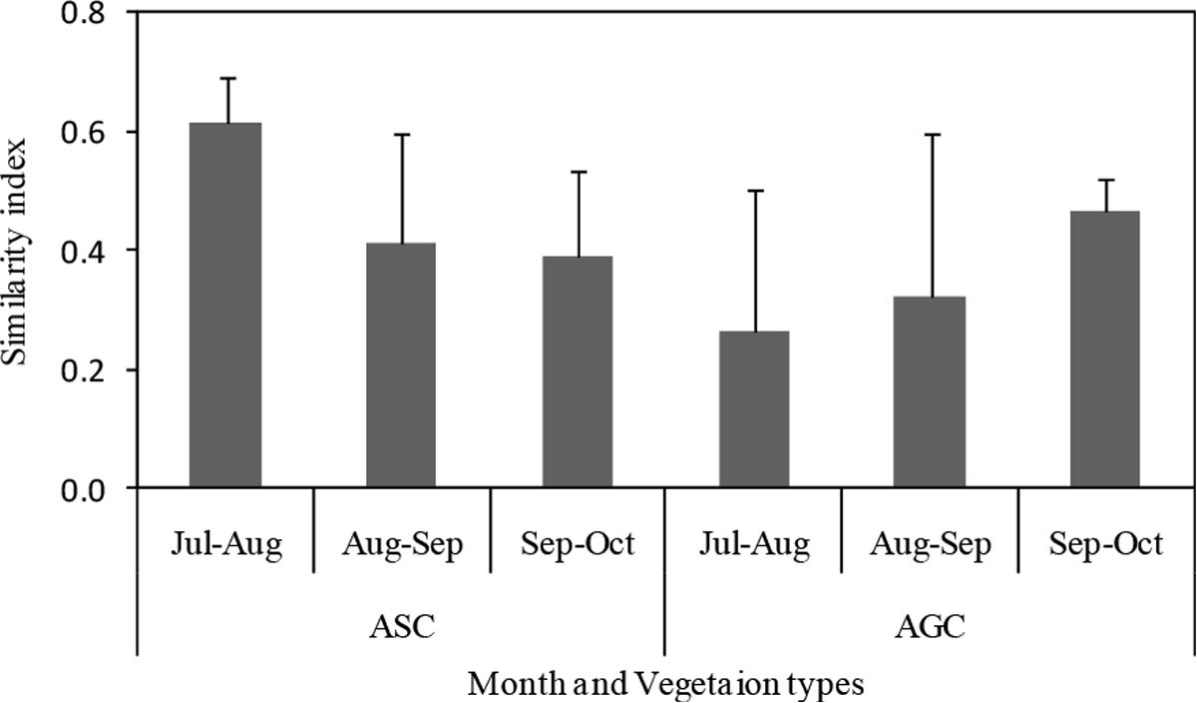
**Similarity between monthly seedlings in ASC and AGC**.

The average similarity index of ASC was 0.47, ranging from 0.39 to 0.61, and the similarity index decreased from July to October; while the average similarity index in AGC was 0.35, ranging from 0.26 to 0.46, with an increase trend (Figure 4).

*L. davurica, D. moldavicaand* and *A. scoparia* appeared each month in ASC, while *Euphorbia humifusa* was found in both of July and August, *Salsola collina* was found in July, August and September, *Poa sphondylodes* and *Potentilla tanacetifolia* were found both in September and October (Table 2). In AGC, species appeared in each month were *L. davurica, S. viciifolia* and *D. moldavicaand*. *Vicia amoena* was found both in July and August. 8 other species appeared both in September and October, except for *L. davurica, S. viciifolia* and *D. moldavicaand*. There were some owerwintering seedlings appeared both in September and October, such as *Cleistogenes squarrosa, Bothriospermun secundum, Viola dissecta* and *A. gmelinii* (Table 2). Compared with AGC, less seedlings species were found in ASC, but the species composition kept relative stable; in contrary, more seedling species were found in AGC, but the composition changed more frequently.

**Table 2:** Seedlings species of dynamic and final density

Types	Specise	Month			Final density (N m^-2^)
		**7-8**	**8-9**	**9-10**	
	*Lespedeza davurica*	+	--	--	2.5
	*Euphorbia humifusa*	--	--		0
	*Dracocephalum moldavicaand*	--	--	--	0.2
ASC	*Salsola collina*	/	--	--	0
	*Artemisia scoparia*	+	--	+	25.2
	*Potentilla tanacetifolia*			/	0.2
	*Poa sphondylodes*			--	0.2
	*Lespedeza davurica*	--	--	--	0.9
	*Sophora viciifolia*	+	--	+	0.5
	*Dracocephalum moldavicaand*	--	+	--	0.3
	*Vicia amoena*	+	--	+	0.7
	*Artemisia gmelinii*	--	+	--	0.3
AGC	*Bothriospermun secundum*			+	0.2
	*Cleistogenes squarrosa*			/	0.2
	*Artemisia hedinii*			--	0.1
	*Viola dissecta*			+	0.3
	*Caragana korshinskii*			+	0.3
	*Ixeris Chinensis*			+	1.6

Note: "+" means increase; "--" means decrease; "/" means stable.

In ASC, density of seedlings decreased after September, except *A. scoparia*. At the end of rain season, two species had a higher density, density of *A. scoparia* seedling was 25.2 n m^-2^ and density of *L. davurica* seedling was 2.5 n m^-2^ (Table 2), which may go through the winter and grow up in the next year. But at the end of growing season, nearly all of the seedling densities in AGC were less than 1 n m^-2^ (Table 2), whether these seedlings would survival in the next spring needs more investigation.

### Similarity between seedlings and vegetation

**Figure 5:**
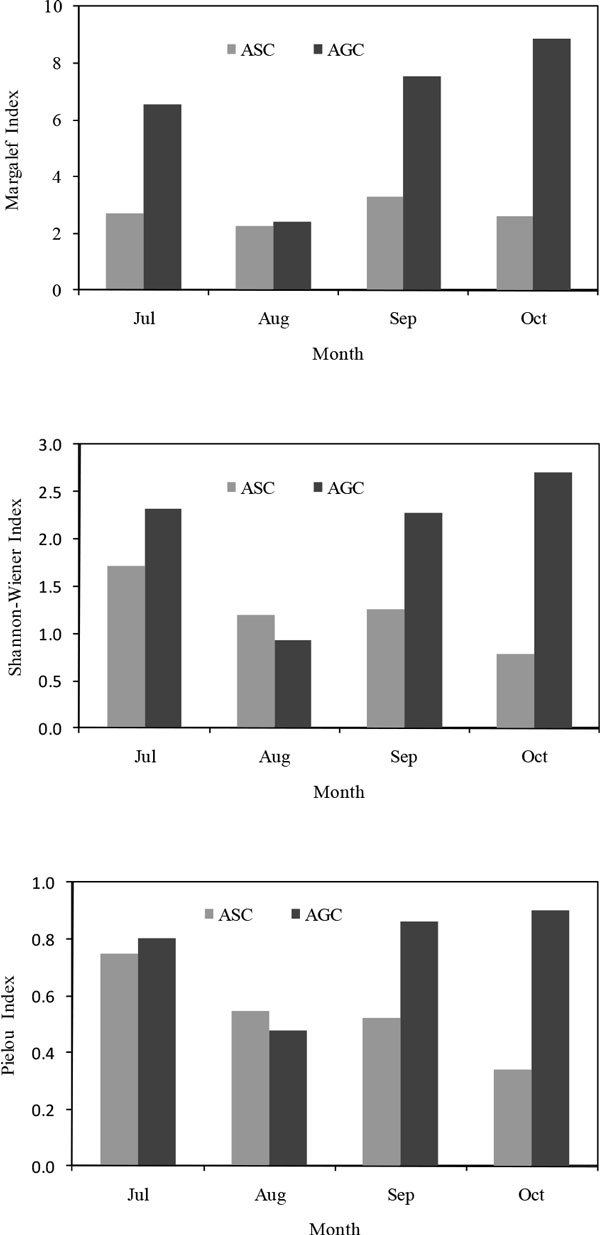
**Similarity between seedlings and mature vegetation in ASC and AGC**.

In ASC, the Sørenson's index of similarity between seedlings and mature vegetation was 0.34, 0.18, 0.26 and 0.32, respectively. And in AGC, the similarity index was 0.30, 0.27, 0.26 and 0.39, respectively (Figure 5). The Sørenson's index of similarity in AGC was higher than that of ASC, but no significant different existed. During the investigation, species in standing vegetation was relative stable, which meant that *A* in Sørenson index formula was relative stable. Then the Sørensen similarity index between seedling and mature vegetation largely depended on seedling species. When a new seed germination and survival, *B* in Sørenson index formula increased, and *W* in Sørenson index formula would have a chance to increase, and the similarity index would rise. Standing vegetation species in AGC were more than that of ASC, and as a result, there would be more kinds of seeds produced and more kinds of seedlings found.

However, the higher similarity index in AGC did not mean seedlings could make more contribution to the community. Seedlings appeared in AGC of each month were *L. davurica, D. moldavicaand* and *S. viciifolia*, they were not the dominant species in AGC. Although the dominant species *A. gmelinii* and *A. giraldii* companied with seedling at the end of the growing season, only 3 *A. gmelinii* seedlings and 2 *A. giraldii* seedlings survived in 10 m^2^. It is difficult for these seedlings to establish a community, in which density of *A. gmelinii* was 3 n m^-2^ and density of *A. giraldii* was 2 n m^-2^. This would be the result of different reproduction types. In ASC, the dominant species *A. scoparia* reproduces only by seed, while the dominant species *A. gmelinii* and *A. giraldii* in AGC reproduces not only by seed but also by rhizomatous [27]. In harsh environment, vegetable propagation reproduction would be the principal way to spreads, which would help vegetative reproduction ramets to beat seed seedlings, then invade and establish successfully [27].

## Conclusions

Compared with *A. gmelinii* + *A. giraldii* communities (AGC), *A. scoparia* communities (ASC) had a higher seedling density and the seedling of dominant species could take approximately 53.2% of the total seedlings. Thousands of seeds existed in soil seed bank and these seeds germinated continuously to keep the seedling density stable. Until the end of the growing season, the survival seedlings density was similar to the standing vegetation. This suggested that *A. scoparia* communities (ASC) may be established by countless seeds germinated and left some lucky seedling to survival.

In contrary, *A. gmelinii* + *A. giraldii* communities (AGC) contained more species in seedlings. But the two dominant species constituted less than 5% of the total seedlings. *A. gmelinii* and *A. giraldii* seedlings could not found in each month, at the end of the growing season, only 2 *A. giraldii* seedlings and 3 *A. gmelinii* seedlings were survival in 10 m^2^, which was lower than the mature vegetation density. This may caused by the scarce seed in soil seed bank and the reproduction strategy. *A. gmelinii* and *A. giraldii* may not establish only by seed germination, and vegetable propagation reproduction would be more important.
